# Adaptation to larval crowding in *Drosophila ananassae* leads to the evolution of population stability

**DOI:** 10.1002/ece3.227

**Published:** 2012-05

**Authors:** Snigdhadip Dey, Joy Bose, Amitabh Joshi

**Affiliations:** Evolutionary Biology Laboratory, Evolutionary and Organismal Biology Unit, Jawaharlal Nehru Centre for Advanced Scientific ResearchJakkur P.O., Bangalore, 560 064, India

**Keywords:** α-Selection, competitive ability, constancy, density-dependent selection, *K*-selection, life-history evolution, persistence, population dynamics

## Abstract

Density-dependent selection is expected to lead to population stability, especially if *r* and *K* tradeoff. Yet, there is no empirical evidence of adaptation to crowding leading to the evolution of stability. We show that populations of *Drosophila ananassae* selected for adaptation to larval crowding have higher *K* and lower *r*, and evolve greater stability than controls. We also show that increased population growth rates at high density can enhance stability, even in the absence of a decrease in *r*, by ensuring that the crowding adapted populations do not fall to very low sizes. We discuss our results in the context of traits known to have diverged between the selected and control populations, and compare our results with previous work on the evolution of stability in *D. melanogaster*. Overall, our results suggest that density-dependent selection may be an important factor promoting the evolution of relatively stable dynamics in natural populations.

## Introduction

Though the connection between crowding and evolution was implicit in the conception of natural selection, the unifying interface between population dynamics and evolutionary genetics was largely unexplored by ecologists and evolutionists during the first half of the 20th century (Mueller and Joshi 2000). The first studies at this interface addressed the effect of ecology on evolution via density-dependent selection, or the notion that genotypic fitnesses could vary as a function of population density (MacArthur 1962; MacArthur and Wilson 1967; Gadgil and Bossert 1970; Roughgarden 1971; Clarke 1972; Asmussen 1983). Many of these studies assumed that life-history related traits affecting intrinsic growth rate (*r*) and carrying capacity (*K*) would tradeoff, and such a tradeoff between realized per capita growth rates at low versus high densities was empirically demonstrated in *Drosophila melanogaster* by Mueller and Ayala (1981a). Around the same time, it was also realized that simple population growth models could yield complex and unstable dynamics, especially if intrinsic per capita growth rates were high (May 1974; May and Oster 1976). Yet, a majority of wild and laboratory populations with high female fecundity were found to show relatively stable dynamics (Hassell et al. 1976; Thomas et al. 1980; Mueller and Ayala 1981b) leading to an increasing interest in proximal and ultimate causes of population stability (reviewed by Mueller and Joshi 2000).

Among the suggested mechanisms for the evolution of population stability were group selection favoring populations with stable dynamics (Thomas et al. 1980; Berryman and Millstein 1989) and individual selection on demographic attributes (Turelli and Petry 1980; Mueller and Ayala 1981b; Stokes et al. 1988; Hansen 1992; Ebenmann et al. 1996). Central to all the arguments for the evolution of stability via individual selection were tradeoffs between life-history traits such as fecundity or maturation rates and juvenile survival (Mueller and Ayala 1981b; Ebenmann et al. 1996), or between demographic parameters such as *r* and *K*, or their analogues (Turelli and Petry 1980; Mueller and Ayala 1981b; Hansen 1992). Density-dependent selection, together with an *r*–*K* tradeoff, thus emerged as one of the most likely factors favoring the evolution of population stability because selection at high density would favor traits that increased *K* that would then reduce *r* as a correlated response, yielding greater stability.

Despite the early theoretical interest in this issue, experimental work on the evolution of stability has been limited as yet to very few studies, most using *D. melanogaster* populations in the laboratory (Mueller 2009). Interestingly, despite considerable work on density-dependent selection and adaptations to crowding in *D. melanogaster* (reviewed by Prasad and Joshi 2003), there has been no experimental support for the evolution of population stability as a result of density-dependent selection. The six *r*- and *K*-selected populations that yielded evidence for a tradeoff between realized growth rates at low versus high density (Mueller and Ayala 1981a) were never examined for population stability per se. A later study of the dynamics of five CU (Crowded as larvae, Uncrowded as adults) populations, selected for adaptations to larval crowding, and their five ancestral UU (Uncrowded as larvae, Uncrowded as adults) controls, aimed at asking whether populations maintained on a destabilizing food regime (LH regime: Low larval and High adult food levels; Mueller and Huynh 1994) would evolve greater stability and, if so, whether the CU populations would evolve stability faster than the UU populations (Mueller et al. 2000). The results of that study showed that although evolution of traits related to competitive ability did occur during the course of the study (Joshi et al. 2003), the sole determinant of stability was food regime (destabilizing or stabilizing) and there was no effect of past selection or a selection × food regime interaction (Mueller et al. 2000).

A couple of studies have identified tradeoffs involving fecundity as the likely cause mediating the evolution of stability in laboratory populations (Stokes et al. 1988; Prasad et al. 2003). A reanalysis of Nicholson's (1957) blowfly data suggested that the evolution of stability over a long period of time in some of the experiments was consistent with inadvertent selection for the ability of female blowflies to lay eggs under severe protein deprivation in some food treatments, and a tradeoff between this ability and both survival and maximal fecundity (Stokes et al. 1988). The clearest evidence yet of the evolution of stability as a by-product of life-history evolution has come from a study of four *D. melanogaster* populations selected for rapid preadult development and their ancestral controls (Prasad et al. 2003). The selected populations had evolved reduced body size, fecundity, and preadult survivorship relative to controls as correlated responses to selection for rapid development (Prasad et al. 2000, 2001; Joshi et al. 2001), and also exhibited greater constancy stability than controls in their population dynamics when maintained on a destabilizing food regime (Prasad et al. 2003; Dey et al. 2008).

In Nicholson's (1957) experiment, the density of the blowfly populations was not explicitly controlled and often the larvae and adults underwent severe crowding. In some of these populations, females with lower minimum protein requirements for egg laying appear to have been favored by selection acting during recurrent episodes of severe adult food limitation. Females with lower minimum protein requirement also had lower maximal fecundity at very low density, a trait likely to translate into lower *r*. Nicholson's (1957) observation is, thus, a potential example of episodic crowding driving the evolution of greater stability via a tradeoff between fecundity at high and low densities. However, this particular explanation of these experimental results is post hoc and, though plausible, does not have direct empirical support. The study of Prasad et al. (2003) is also difficult to interpret as an example of density-dependent selection causing the evolution of stability as the selected populations were kept at a constant moderately low density, with no possibility for inadvertent episodes of crowding. Thus, despite the implication from theory that density-dependent selection is likely to be an important factor driving the evolution of population stability, there is no clear evidence for adaptation to crowding actually resulting in the evolution of more stable dynamics in laboratory or wild populations.

In this paper, we present the first clear experimental evidence for the evolution of greater population stability as a consequence of density-dependent selection in laboratory populations of *D. ananassae* subjected to selection for adaptation to larval crowding. We show that the selected populations evolve both greater constancy and persistence than ancestral control populations after approximately 70 generations of selection at high larval density, and that the evolution of stability appears to be mediated by the evolution of greater *K* and reduced *r* in the selected populations. We also propose an explanation for how increased realized growth rates at high densities can result in stability even if unaccompanied by a reduction in maximal growth rates. We test this explanation using simulations based on three standard models of population growth. Overall, our results suggest that density-dependent selection may be a fairly common factor promoting stability in natural populations.

## Materials and Methods

### Experimental populations

Eight large (*N*∼ 1800) laboratory populations of *D. ananassae*, reared on cornmeal medium and maintained on a 21-day discrete generation cycle at ∼25°C and constant light (LL) were used in the present study. Four of these populations (*AB*_1–4_) served as controls and were maintained at moderate larval densities (∼70 eggs in 6-mL medium). The other four populations (*ACU*_1–4_) were each derived from one of the controls and were selected for adaptation to larval crowding by maintaining them at high larval density (550–600 eggs in 1.5-mL medium). For both sets of populations, eggs were placed at the required density in glass vials (2.4 cm diameter × 9 cm height) and eclosed adults were collected into Plexiglas cages (25 × 20 × 15 cm^3^) containing a petridish of cornmeal medium and a wad of moist cotton wool to maintain high humidity. In the *AB* populations, adults were collected into cages on the 12th day after egg collection. In the *ACU* populations, adults were collected into cages once every day after eclosion began till day 18 after egg collection. All adults in cages were supplied cornmeal medium with a generous smear of live yeast–acetic acid paste for three days prior to egg collection for initiating the next generation. These populations were first described in detail by Sharmila Bharathi (2007), and at the time the time-series experiment described below was set up, the *ACU* populations had undergone 69 generations of selection, and showed significantly higher competitive ability than the control *AB* populations (Archana 2010).

## Time series experiment

To study the population stability of the selected and control populations under common conditions, eight replicate small (single vial) populations were set up from each of the eight *AB* and *ACU* populations, resulting in 64 single vial populations. All these vial populations were initiated by collecting exactly 30 eggs in approximately 1-mL of cornmeal medium per vial. The populations were all subsequently maintained on a 21-day discrete generation cycle (following the general procedure of Sheeba and Joshi 1998) with no explicit control exerted on the population densities during the course of this 25-generation long experiment. Once the adults in these single vial populations started eclosing, they were transferred daily to adult collection vials containing approximately 4-mL cornmeal medium. Adult collection was continued till the 18th day from egg collection, by which time all adults from viable pupae would have typically eclosed. Every alternate day, the adults were transferred to fresh adult collection vials. Extreme care was taken during all vial-to-vial transfers to prevent any possible loss of flies. From the 18th to 21st day after egg collection, adults were also supplied with a dab of live yeast–acetic acid paste that boosts female fecundity (Chippindale et al. 1993). This kind of nutritional regime with low levels of larval food and yeasting at the adult stage has been shown to produce high amplitude two-point oscillations in population size in *D. melanogaster* (Mueller and Huynh 1994; Sheeba and Joshi 1998; Mueller et al. 2000). On the 21st day after egg collection, the number of adults (population size) of each vial was censused and recorded. The adults were then transferred to fresh egg collection vials with 1 mL of cornmeal medium and discarded after 24 h; the eggs laid by them started the next generation. In case an extinction (a population having not even one male and one female) occurred in any vial, the population was restarted with two males and two females from back up vials that were run in parallel with the experimental vials. This maintenance protocol was continued for 25 generations and the resulting time series of adult numbers were subjected to further analyses to determine stability.

### Data analysis

#### Measures of population stability

Constancy stability of a population is inversely related to the magnitude of fluctuation in numbers it shows across time (Grimm and Wissel 1997). We used two statistics to measure the constancy of the experimental populations, viz. fluctuation index (henceforth, FI; Dey and Joshi 2006) and coefficient of variation (henceforth, CV). *FI* is the mean one-generation change in population size, scaled by average population size of the time series. Thus, *FI* is calculated as 

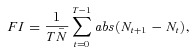

where *T*= length of the time series in generations, 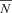
= average population size over the *T* generations, and *N_t_*= size of the population at generation “*t.*”

Persistence stability of a population was taken to be inversely related to the number of extinctions it suffered during the course of the experiment. Following Dey et al. (2008), extinctions in consecutive generations in the same single-vial population were treated as a single extinction event, as such consecutive extinctions are not independent.

### Density-dependent growth rates and demographic parameters

In order to estimate the intrinsic growth rate (*r*) and equilibrium population size (*K*), two indirect approaches were followed. First, following Joshi et al. (2001), the realized population growth rates (*N_t_*_+1_/N*_t_*) for each single-vial population at low (*N_t_*≤ 30) and high (*N_t_* > 60) densities were considered to reflect the magnitude of *r* and *K*, respectively. As long as *r* values are not very different between populations, greater growth rate at high densities can be assumed to reflect a greater *K*. This analysis was repeated for different cut-off values for defining “low” and “high” density, to ensure robustness of the conclusions drawn. In the second approach, regression lines were fitted to the scatter plot of Log*N_t_*_+1_/Log*N_t_* versus Log*N_t_* for each single-vial population, and the Y- and X-intercepts and slope of the regression line were considered as surrogate measures of *r*, *K*, and the sensitivity of population growth rate to increasing density, respectively.

### Tests of significance

Measures of population stability (both constancy and persistence), density-dependent growth rates, and demographic parameters were subjected to separate mixed-model analyses of variance (ANOVAs), wherein block (i.e., population level replication in the selection experiment, reflecting ancestry) was treated as a random factor with four levels and was crossed with the fixed factor selection regime. The eight single-vial populations for each block × selection regime combination (*AB*_1–4_ and *ACU*_1–4_) provided replicate measures of stability/growth rate for each of the eight experimental populations. All statistical analyses were done using STATISTICA for Windows version 5.0 (StatSoft 1995).

### Simulations

**Figure 1:**
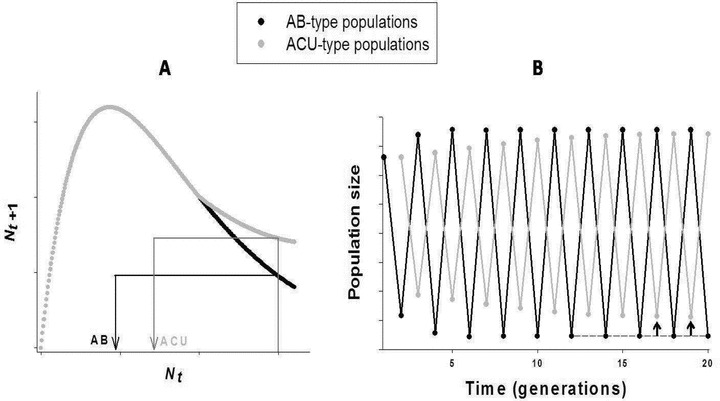
An explanation for greater constancy and persistence in populations with higher growth rates at high densities. A schematic return map of control *AB*-type populations and crowding adapted *ACU*-type populations with elevated realized growth rates at high densities without an *r*–*K* tradeoff (A). The *ACU*-type populations tend to fall to relatively higher (less low) values of *N_t_*_+1_ for a given high value of *N_t_*, compared to the *AB*-type populations. Consequently, the *ACU*-type populations hit very low population sizes substantially less frequently than *AB*-type populations, and also show reduced amplitude density-dependent fluctuations in population size, resulting in both higher constancy and persistence than the *AB*-type populations even without an *r*–*K* tradeoff (B).

We also carried out simulations based on three commonly used models of population growth to test a simple proposed explanation of how high population growth rates at high density might yield increased stability even without a concomitant reduction in population growth rates at low density (Fig. 1). The three models we used were the Ricker (Ricker 1954), Hassell (Hassell et al. 1976), and Theta-Ricker (Thomas et al. 1980) models. The simulations were carried out over a range of bifurcation parameter values for the respective models that would yield gross dynamic behavior involving moderately large and semi-regular fluctuations between low and high population sizes, as shown by our experimental populations. Beyond that, the simulations did not attempt to mimic the experiment. They extended the implications of the experimental study by exploring the possibility of enhanced stability via increased growth rates at high density (e.g., due to higher *K*) alone, even without an *r*–*K* tradeoff as seen in the experiment. Part of the reason for doing the simulations was because the reduction in *r* in the selected populations, though large, was statistically not significant (see Results).

#### Ricker model


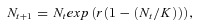
1
where *N_t_*= population size at generation “*t*,”*r*= maximal per capita growth rate, and *K*= carrying capacity/equilibrium population size. The gross dynamic behavior of the Ricker model depends solely on the “*r*” values, with stable point behavior for *r* < 2, periodic dynamics for 2 < *r* < 2.692, and chaotic dynamics for *r* > 2.692 (May and Oster 1976).

#### Theta-Ricker model


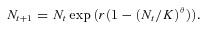
2
Equation (2) is a modification of Equation (1) through incorporation of one more parameter (θ), which reflects density sensitivity. For θ >1, Equation (2) can show chaos depending on the values of *r* (Getz 1996).

#### Hassell model


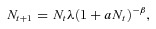
3
where, λ is the finite rate of increase, and *a* and β are density feedback constants. The stability analysis of Equation (3) has been reported in Hassell (1975) and Hassell et al. (1976).

### Implementation of the simulations

For all the simulations, we considered control (uncrowded; henceforth, *AB*-type) and the selected (crowded at larval stages; henceforth, *ACU*-type) populations, but without an *r*–*K* tradeoff. Thus, the *ACU*-type populations were allotted a growth rate advantage (a constant value added to the basal realized values; 0.2 for simulations using Equation 1 and 2, and 4 for Equation 3) at higher population densities (for *N_t_* > 40 in case of Equation 1 and 2, and for *N_t_* > 120 for Equation 3), while keeping the growth rates same for the two types of population at lower population densities. Time series were generated by iterating the models for 100 generations, and the *FI* values were calculated to study the stability of the *ACU*-type and *AB*-type populations. All the simulations were made stochastic by adding a uniform random variable to the growth rates each generation, and 10 replicate time series were generated in all the cases. Since our experimental populations showed moderately large and semi-regular fluctuations between low and high population sizes, we focused only on values of the bifurcation parameters that fell in the periodic zone of these three models. To explore the robustness of the results, the simulations were carried out for different constant values added to the growth rates of *ACU*-type populations and by setting up different threshold population sizes beyond which the growth rate advantage was implemented.

## Results

### Population stability

**Figure 2:**
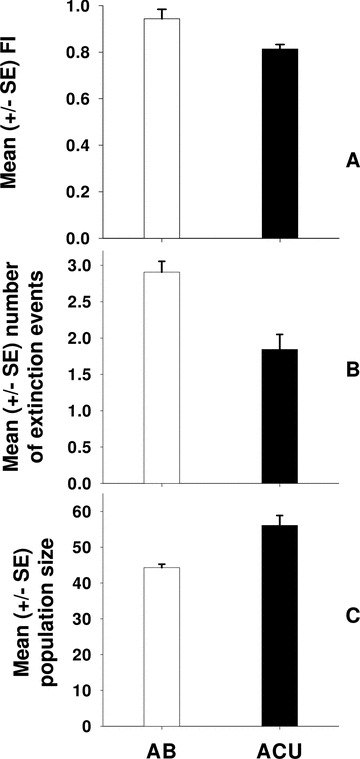
Experimental results: stability. *ACU* populations showed significantly higher constancy (A) and persistence (B) than the control *AB* populations, indicating evolution of enhanced stability as a result of adaptation to larval crowding. (C) *ACU*s also maintained higher population sizes on average than the control *AB*s.

The results of the mixed-model ANOVA showed that the *ACU* populations had evolved enhanced constancy stability as a by-product of adaptation to larval crowding. The mean *FI* value in the *ACU* populations was significantly (*F*_1,3_= 11.794; *P*= 0.041) lower than in the *AB* controls (Fig. 2A). The same pattern was seen in case of the *CV*, with the *ACU* populations showing lower mean *CV* than the *AB* populations, although in this case the difference was marginally nonsignificant (*F*_1,3_= 7.3; *P*= 0.074).

The ANOVA results also showed that the *ACU* populations had evolved greater persistence than the *AB* controls, as the mean number of extinction events over 25 generations was significantly (*F*_1,3_= 22.102; *P*= 0.018) lower in the *ACU* populations, compared to the *AB* controls (Fig. 2B).

### Average population size

The mean average population size in the *ACU* populations (56.3) was about 27% higher than in the *AB* populations (44.3) (Fig. 2C), and the ANOVA revealed that this difference was significant (*F*_1,3_= 27.217; *P*= 0.014). Thus, after being selected for adaptation to larval crowding for 69 generations, the *ACU* populations were able to maintain higher population sizes on average in the single-vial cultures.

### Density-dependent growth rates and demographic parameters

**Figure 3:**
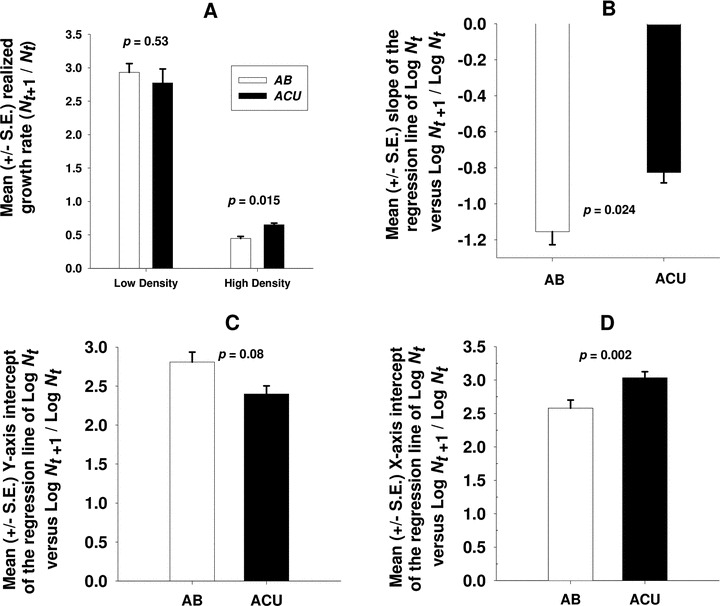
Experimental results: density-dependent growth rates. (A) The realized growth rates (*N_t_*_+1_/*N_t_*) of the two types of populations were not significantly different at low densities (*N_t_* < 30) but at high densities (*N_t_* > 60), *ACU* populations had significantly higher realized growth rates than the *AB* populations. (B) Realized growth rates of *ACU*s were significantly less sensitive to density than those of the *AB*s. (C) The *ACU*s showed lower intrinsic growth rates than *AB*s, though the difference was marginally nonsignificant. (D) The *ACU*s had significantly higher equilibrium population size than the *AB*s. The *P* values indicate the level of significance obtained from the mixed-model analysis of variance (ANOVA).

At low population densities (*N_t_*≤ 30), the mean realized growth rates of *AB* and *ACU* populations did not differ significantly (*F*_1,3_= 0.504; *P*= 0.529). However, the power of this analysis is likely to be low because the realized growth rates are measured directly from the time series and are therefore subject to noise due to the variation in *N_t_* values as also *N_t_*_-1_ values (Prout and McChesney 1985). At high population densities (*N_t_* > 60), *ACU* populations had significantly higher growth rates than the *AB* controls (*F*_1,3_= 25.845; *P*= 0.015) (Fig. 3A). This pattern of results of the *ACU* populations showing significantly higher mean realized growth rates than the *AB* controls at high densities, but similar growth rates at low densities, was seen across many analyses in which the cut-off values defining the “low” and “high” densities were varied (data not shown).

The results obtained from the second analysis of density-dependent growth rates and demographic parameters yielded evidence for a tradeoff between *r* and *K* in these populations. The mean slope of the regression line fitted to the scatter plot of the log-transformed population growth rates (Log*N_t_*_+1_/Log*N_t_*) versus population densities (Log*N_t_*) was significantly more negative in the *AB* populations (*F*_1,3_= 17.963; *P*= 0.024), indicating reduced sensitivity of realized growth rates to density in the *ACU* populations that had adapted to larval crowding (Fig. 3B). The mean Y-intercept of the regression lines was about 12% higher in the *AB* than the *ACU* populations (Fig. 3C), but the difference was marginally nonsignificant (*F*_1,3_= 6.907; *P*= 0.078). The *AB*–*ACU* difference, however, is sufficiently large to constitute a biologically meaningful fitness difference between the selected and control populations at very low density. Power calculations showed that the mean Y-intercept of the *AB* populations would have to be over 20% higher than that of the *ACU* populations for the difference to be picked up as being significant at the 0.05 level, supporting the interpretation that *r* in the *ACU* populations was probably lower than that in the *AB* controls. The mean X-intercept of these regression lines was significantly greater in the ACU populations (*F*_1,3_= 99.945; *P*= 0.002) indicating substantially higher *K* values in the crowding adapted populations as compared to the controls (Fig. 3D). Thus, our results, like previous observations on *D. melanogaster* (Mueller and Ayala 1981a), at least strongly suggest the existence of a tradeoff between realized population growth rates at low and high densities.

### Simulation results

**Figure 4:**
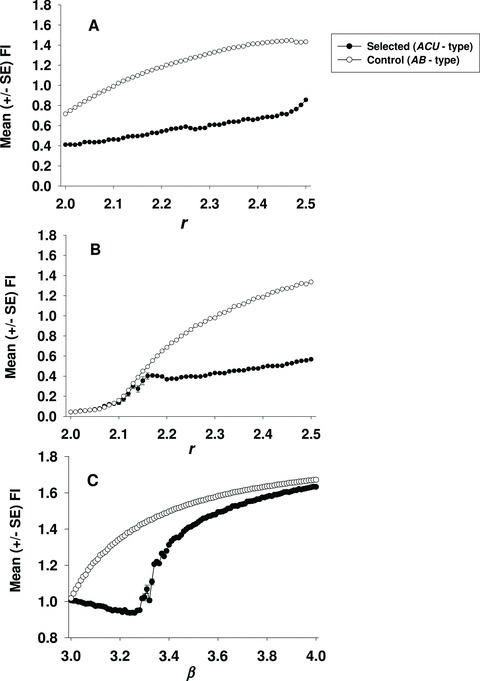
Simulation results on constancy stability of AB-type and *ACU*-type populations. (A) Ricker model: *ACU*-type population received growth increment of 0.2 for *N_t_* > 40. (B) Theta-Ricker model: θ= 0.9. Growth rate increment of 0.2 was given to *ACU*-type population at *N_t_* > 40. (C) Hassell model: *a*= 0.01, λ= 30. *ACU*-type population received a growth rate increment of 4 at *N_t_* > 120.

In our simulations, the *ACU*-type populations showed lower *FI* values, implying greater constancy, and this pattern was consistently seen in simulations with all the three models (Fig. 4). On average, the troughs in population size reached by the *ACU*-type populations were also higher than those attained by the *AB*-type populations (data not shown), implying the possibility of greater persistence. The simulations thus supported the logic of our proposed explanation that populations possessing a growth rate advantage at high population densities will exhibit enhanced constancy and persistence because they will have a lower probability of reaching very low population sizes, essentially reducing the amplitude of population size fluctuations in addition to setting a floor for population size (Fig. 1). Thus, the evolution of enhanced *K* could in principle yield greater constancy and persistence stability even in the absence of an *r*–*K* tradeoff.

## Discussion

The results of this study provide clear evidence for the evolution of greater persistence and constancy stability as a result of adaptations to larval crowding in laboratory populations of *D. ananassae*. Contra Dey et al. (2008), these results thus suggest that constancy and persistence can evolve in tandem. This is the first experimental result linking density-dependent selection to the evolution of population stability via elevated growth rates at high density, often a correlate of increased *K* as seen in the *ACU* populations (Fig. 3D). The experimental data indicate that *r* in the *ACU* populations is likely to have decreased relative to the *AB* controls (Fig. 3C). Moreover, the results from the simulations clearly show that higher population growth rates at high population densities yield both greater constancy (lower FI) and persistence (higher troughs in population size, on average), even in the absence of a correlated reduction in the maximal population growth rate, *r* (Fig. 4), at least in populations undergoing semi-regular oscillations in numbers. Given that adaptation to crowding via increased *K* can thus lead to greater stability with or without a concomitant reduction in *r*, it is likely that density-dependent selection may often lead to the evolution of greater population stability. We now discuss our experimental results in the context of the traits known to have evolved in the crowding adapted *ACU* populations, and compare these results with those of an earlier study showing the evolution of greater constancy but not persistence stability in *D. melanogaster* populations subjected to selection for rapid development (Prasad et al. 2003; Dey et al. 2008). We conclude by discussing the implications of these studies for the way in which we think about density-dependent selection.

We next address the issue of what traits might be driving the higher growth rate at high but not low densities in the *ACU* populations (Fig. 3A). Previous studies (Sharmila Bharathi 2007; Archana 2010) have shown that, relative to the *AB* controls, the crowding adapted *ACU* populations exhibit greater preadult competitive ability, decreased preadult development time at both low and high larval densities, and decreased preadult survivorship at low densities but increased preadult survivorship at high densities. Fecundity and dry weight at eclosion of flies from the *ACU* populations are not significantly different from the *AB* controls after rearing at either high or low larval density, but there is a tendency in repeated assays for *ACU* flies to be slightly heavier at eclosion than controls. In contrast to earlier studies on crowding adapted *D. melanogaster* (Joshi and Mueller 1988, 1996), larval feeding rates in the *ACU* populations are not significantly different from controls, although the *ACU* populations do show higher foraging path lengths, pupation heights, and ammonia tolerance than controls, findings consistent with earlier results using *D. melanogaster* (reviewed by Prasad and Joshi 2003). There is also some suggestive evidence for possible reduced critical minimum food requirement for pupation in the *ACU* populations (Sharmila Bharathi 2007); if indeed the case, this would also be in contrast to the finding of increased minimum food requirement in crowding adapted *D. melanogaster* populations (Mueller 1990; Joshi and Mueller 1996).

One major difference between the *ACU* populations and the crowding adapted populations of *D. melanogaster* studied earlier (Mueller 1990; Joshi and Mueller 1996), thus appears to involve the mechanisms by which adaptation to larval crowding was achieved. The *D. melanogaster* populations became more competitive primarily through increased larval feeding rate and urea/ammonia tolerance (Joshi and Mueller 1996; Shiotsugu et al. 1996), rather than by the classic *K*-selection mechanism (MacArthur and Wilson 1967) of greater efficiency of conversion of food to biomass (Mueller 1990; Joshi and Mueller 1996). Density-dependent selection in the crowding adapted *D. melanogaster* populations is thus better viewed in terms of having increased competitive ability directly (*α*-selection) rather than affecting competitive ability through selection acting to increase *K* (Joshi et al. 2001). Such evolutionary increases in competitive ability without increased *K* have been theoretically shown to be possible (Assmussen 1983; Mueller 1988). To the extent that evolutionary increases in competitive ability due to crowding adaptation are attained through traits affecting primarily the frequency-dependent aspect of competition (e.g., increased larval feeding rate), they are unlikely to affect the values of summary demographic parameters (e.g., *r* and *K*) and, therefore, are also unlikely to affect population stability. Evolution in the *ACU* populations, on the other hand, appears to have been closer to the canonical notion of *K*-selection (MacArthur and Wilson 1967), involving increased ammonia tolerance and, very possibly, greater efficiency of conversion of food to biomass, leading to decreased sensitivity of population growth rates to high density and an increase in *K* accompanied by a reduction in *r* (Fig. 3B–D). Earlier studies on the *ACU* and *AB* populations also showed a tradeoff between preadult survivorship at low and high larval density (Sharmila Bharathi 2007), consistent with an *r*–*K* tradeoff in these populations.

Interpreting these findings in terms of the “effectiveness” and “ tolerance” aspects of competitive ability (sensu Joshi et al. 2001), we can say that reduced development time (with a tendency to greater weight at eclosion) and minimum food requirement together with increased pupation height and ammonia tolerance are likely to enhance the “tolerance” of *ACU* populations to the inhibitory effects of a competitor on their population growth rates. On the other hand, increased larval foraging path lengths are unlikely to cause more than a slight increase in the “effectiveness” of *ACU* populations at reducing the population growth rate of a competitor, given that larval feeding rates in the *ACU* and *AB* populations do not differ significantly. The “effectiveness” component of competitive ability is the one that is more commonly strongly frequency dependent, supporting the view that the *ACU* populations have adapted to crowding through means closer to the canonical notion of *K*-selection.

The difficulty of interpreting studies on population stability and adaptation to crowding in *Drosophila* in terms of *K*-selection or *α*-selection is underscored by comparing the results of the present experiment with earlier work on crowding adapted (Mueller et al. 2000) and rapid developing (Joshi et al. 2001; Prasad et al. 2003) populations of *D. melanogaster*. The crowding adapted CU populations used by Mueller et al. (2000) did not show enhanced stability or any change in surrogates of *r* or *K*, compared to controls, and as discussed earlier appear to have evolved greater preadult competitive ability through *α*-selection rather than *K*-selection. On the other hand, relative to controls, the rapid developing FEJ (Faster developing, Early reproducing, derived from Joshi baseline populations) populations showed reduced preadult competitive ability (Shakarad et al. 2005), urea tolerance (Joshi et al. 2001), and larval feeding rates (Prasad et al. 2001), but enhanced constancy and population growth rates at high density (Prasad et al. 2003; Dey et al. 2008), as well as reduced body size, preadult survivorship, and fecundity (Prasad et al. 2000). There was also suggestive evidence for a reduction in minimum food requirement in the FEJ populations (Prasad et al. 2001). The FEJ populations were selected for rapid preadult development and relatively early reproduction but were maintained at the same larval density as their ancestral controls (Prasad et al. 2000). Yet, the pattern of evolution of traits in the FEJ populations is somewhat intermediate between the canonical expectations of *α*-selection and *K*-selection sensu Joshi et al. (2001). The FEJ populations appear to have undergone a reduction in both “effectiveness” and “tolerance” components of *α*, via reduced larval feeding rates and urea tolerance, and an increase in *K* via the reduction in body size and minimum food requirement (Joshi et al. 2001). The *ACU* populations used in this study, as discussed earlier, seem to have evolved competitive ability through traits resulting in a clear increase in *K*, possibly through greater efficiency of conversion of food to biomass, a classic *K*-selected trait.

The consideration of results from all these studies together suggests that it is more meaningful to focus on the actual traits that evolve under different selection regimes, and how they might affect equilibrium size (*K*), competitive ability (*α*), and density-dependent population growth rates, if trying to understand how population stability might evolve as a correlated response to selection. Notions of *K*-selection and α-selection focus attention on population-level characteristics whereas selection acts primarily on traits expressed at the level of individuals. As the preceding discussion shows, both *K* and *α* can in principle be affected by the suite of traits evolving in response to a given selection regime, regardless of whether the selection is along a density axis. The critical issue, therefore, is not to label the type of selection using these population parameters but rather to understand how the individual-level traits that have evolved affect density-dependent population growth rates, especially at high density, and the degree to which these effects are frequency dependent. The comparison of the present results with those of Mueller et al. (2000) and Prasad et al. (2003) on *D. melanogaster* suggests that when adaptation to crowding involves an increase in population growth rates at high density through evolutionary changes in traits whose effects on population growth rates are not strongly frequency dependent, population stability is also likely to evolve as a correlated outcome. If, on the other hand, adaptation to crowding occurs largely through traits whose effects on population growth rates are strongly frequency dependent, as in the case of the CU populations (Mueller et al. 2000), then summary demographic parameters and population stability are unlikely to be affected. Populations in nature can be expected to evolve a variety of traits under selection to adapt to crowding, and it is likely that many of these traits will have non-frequency-dependent effects on population growth rates. Thus, the observation that higher population growth rates at high density can result in greater constancy and persistence stability even in the absence of a tradeoff with population growth rates at low density (Fig. 4) suggests that density-dependent selection may be an important factor promoting the evolution of stable dynamics in natural populations.
